# Network pharmacology and bioinformatics approach to unravel the mechanism of Xiao-chai-hu-tang herbal formula in tinnitus treatment

**DOI:** 10.1016/j.heliyon.2024.e37584

**Published:** 2024-09-10

**Authors:** Shihan Liu, Xintong Zhou, Lingli Zhang, Wenlong Luo

**Affiliations:** a Department of Otorhinolaryngology, the Second Affiliated Hospital of Chongqing Medical University, Chongqing, China; b College of First Clinical Medicine, Shandong University of Traditional Chinese Medicine, Jinan, China; c Department of Otorhinolaryngology, Central Hospital Affiliated to Chongqing University of Technology, Chongqing, China

**Keywords:** Xiao-chai-hu-tang, Network pharmacology, Mendelian randomization, Molecular docking, Tinnitus, HIF1A

## Abstract

**Background:**

Tinnitus treatment remains a global challenge, and current therapeutic approaches are still controversial. This study aims to elucidate the potential mechanisms of Xiao-Chai-Hu-Tang (XCHT) in treating tinnitus through the analysis of network pharmacology, mendelian randomization and molecular docking, and molecular dynamics simulation analysis. We hope to contribute to the research on the target of action of traditional Chinese medicine and exploration of the mechanism of tinnitus.

**Methods:**

We utilized network pharmacology to screen potential targets of action of XCHT on tinnitus. Mendelian randomization was employed to determine the causal relationship between potential targets of action and tinnitus. Finally, molecular docking and molecular dynamics simulation with clear targets and the combination of the active ingredient in effectiveness.

**Results:**

Through network pharmacology, we identified 38 potential targets of action. Mendelian randomization analysis revealed that HIF1A (OR [95 % CI] = 0.78 [0.65, 0.94], P = 0.008) and CCND1 (OR [95 % CI] = 1.22 [1.00, 1.49], P = 0.04) exhibited significant results with tinnitus. Molecular docking and molecular dynamics simulation of HIF1A and active ingredients demonstrated good binding efficacy.

**Conclusion:**

HIF1A may play a key role in the treatment of tinnitus by XCHT, which may play a certain protective role in tinnitus patients and may inhibit the occurrence and development of tinnitus. However, the specific mechanism and effect need to be further studied and verified.

## 1 Introduction

Tinnitus, a common disorder in otolaryngology, refers to the perception of sound within the ear in the absence of external electrical or acoustic stimulation [1]. Statistics indicate that approximately 10 %–15 % of individuals are affected by tinnitus for a significant period [2]. The pathogenesis of tinnitus is complex and remains poorly understood, possibly associated with cochlear abnormalities [3]. Declining high-frequency hearing is a major risk factor for tinnitus [3], which often leads to symptoms such as poor concentration, cognitive impairment, irritability, and insomnia, significantly affecting daily life [4]. Currently, there are few effective treatments for tinnitus through medication or surgery, and no specific therapy for tinnitus is considered satisfactory for all patients, mainly due to its complex etiology and unclear mechanisms [5]. Consequently, an increasing number of tinnitus sufferers are turning to traditional Chinese medicine (TCM) for help, as TCM offers unique insights into the mechanisms underlying tinnitus and guides the use of medications for its treatment.

In TCM, Xiao-chai-hu-tang (XCHT) has been used to treat tinnitus for nearly a millennium. XCHT is thought to help restore the body state of balance by modulating stress responses, reducing inflammation, and supporting overall well-being. It is also believed to soothe the nervous system and alleviate emotional distress, which may contribute to the relief of tinnitus symptoms [6,7]. Clinical observations suggest that modern lifestyle factors, such as increased stress and emotional disturbances, may contribute to conditions like tinnitus [4]. The mental state of imbalance is thought to manifest as symptoms such as tinnitus, although the precise physiological mechanisms linking stress, emotional state, and tinnitus require further scientific investigation. In the context of TCM, certain herbal formulations are thought to address the underlying imbalances associated with tinnitus. XCHT includes ingredients such as Radix Bupleuri, Scutellaria baicalensis, Ginseng, Licorice, Ginger and Jujube kernel, Pinellia, and is thought to act on several physiological systems. The use of Radix Bupleuri in the treatment of mental health issues such as anxiety and depression is highly significant [[8], [9], [10]]. Additionally, Pinellia Tuber, known for its anti-inflammatory properties [11], could potentially aid in the management of tinnitus. Furthermore, Ginseng and Jujube, which are beneficial for enhancing blood circulation [12,13], may also contribute to alleviating the symptoms of tinnitus.

**Fig. 1:**
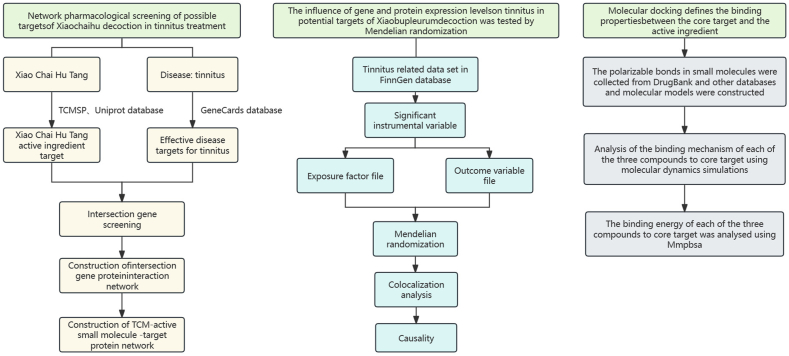
Flow chart of this study.

TCM is involved in the treatment of diseases through multiple pathological targets and pathways [14]. The mechanism of action of XCHT in treating tinnitus is currently unclear. Network pharmacology is used to find potential key targets for the action of drugs on diseases through the synergistic action of multiple components, channels, and targets [15]. Mendelian randomization (MR) is a powerful statistical method for exploring causal relationships between targets and diseases [16], and has played a key role in drug target research in recent years [17]. This method uses common genetic variations to clarify causal relationships between the two, avoiding the confounding and bias factors in “natural randomized trials”. Molecular docking studies the interaction between molecules and predicts their binding patterns and affinities [18]. In addition, molecular dynamics simulations provide insights into the dynamic binding characteristics and conformational changes of drug-target interactions over time [19]. The unique characteristics of Chinese herbal medicine, with multiple targets, pathways, and links, are in line with the concepts of network pharmacology, MR, and molecular docking. Therefore, this study aims to identify the pharmacological potential targets of XCHT in treating tinnitus from the perspectives of network pharmacology, MR and molecular docking, and molecular dynamics simulations (Fig. 1).

## 2 Materials and methods

In this study, the network pharmacology approach was used to screen the potential targets of XCHT in the treatment of tinnitus, and the core targets with significant effects were further screened by MR analysis. Finally, through the molecular docking and molecular dynamics simulation, evaluation targets of potential efficacy and mechanisms of these core.

### 2.1 Target screening of active ingredients in traditional Chinese medicine compound

The traditional Chinese Medicine Systems Pharmacology Database and Analysis Platform (TCMSP) database (https://old.tcmsp-e.com/tcmsp.php) was searched for active ingredients of XCHT [20]. Oral bioavailability (OB) refers to the extent and rate at which a drug is absorbed into systemic circulation after oral administration [21]. Drug-likeness (DL) is a chemical qualitative characteristic that is commonly used in the early stages of drug discovery [22]. Active ingredients were screened based on oral bioavailability (OB ≥ 30 %) and drug-likeness (DL ≥ 0.18). All target proteins of each active ingredient were input into the Uniprot database (https://www.uniprot.org/) for corresponding gene matching, with the species set as “Homo sapiens” [23]. The UniProt database provides detailed data on protein sequence, structure, function, and related biological information.

### 2.2 Screening of potential disease targets

GeneCards is a comprehensive biological information database, it provides a wide range of information about human genes. The GeneCards database (https://www.genecards.org/) was searched [24], with “tinnitus” as the keyword. Targets with a relevance score >1 were selected as our effective disease targets, and duplicate targets were removed.

### 2.3 Construction of Cerbal medicine-active ingredient-target network analysis and matching of related overlapping targets

Cytoscape is a powerful bioinformatics tool that enables us to visually explore and analyze complex biological networks through a graphical interface. Cytoscape 3.9 software was used to graph the herbal medicine, active ingredients, and corresponding targets of XCHT [25], constructing a herbal medicine-active ingredient-target network analysis graph. The core active ingredient targets of XCHT intersect with the tinnitus disease targets to produce a comprehensive set of composite targets. We have visualized this composite target set using a VENN diagram.

### 2.4 Overlapping results of targeted active ingredients in traditional Chinese medicine compounds and potential disease targets

The STRING database is a widely used resource that provides a comprehensive view of associations between proteins, both direct (physical) and indirect (functional) interactions. The overlapping targets were imported into the STRING database for analysis, setting the species to “Homo sapiens” and the minimum required interaction score to a medium confidence level (0.4). This threshold balances the specificity and sensitivity of the results, obtaining a protein-protein interaction (PPI) network diagram [26], Simultaneously, in Cytoscape 3.9 software, the overlapping genes were analyzed using the cytohubba tool [27], selecting the top 5 genes in terms of relevance using the MCC algorithm as key targets. The MCC algorithm is a method used to identify key nodes in a network that often correspond to important biological functions in biological networks.

### 2.5 Study design

We used MR to test the effects of gene and protein expression levels of potential targets of XCHT in treating tinnitus. After determining causal effects MR evidence, we conducted colocalization analysis to confirm that exposure and outcome were regulated by the same causal variant.

### 2.6 Dataset description and MR analysis

The diagnosis of tinnitus is based on the tenth edition of the International Classification of Diseases (ICD-10), with the diagnostic code H93.1. In ICD-10, the H93.1 code is used to indicate tinnitus, a symptom of hearing buzzing, ringing, or other sounds in the ear. We used the GWAS dataset of tinnitus from the FinnGen database [28], which included data from 3502 cases and 196,592 European ancestry controls. We performed eQTL analysis on potential drug action genes, with SNP sources for genetic instruments from comprehensive European GWAS [29]. Gene symbols were converted to ENSEMBL IDs for consistency. We set the eQTL P-value threshold to 5 * 10^-8. SNPs associated with potential genes were extracted from the tinnitus GWAS dataset as MR analysis tool variables. Finally, we used the TwoSampleMR package for MR. If only one eQTL was available for a given target, we used the Wald ratio. When two or more genetic instruments were available, the inverse variance weighted MR (MR-IVW) was applied. MR analysis requires that SNPs are not directly related to outcomes. We used the online website PhenoScanner (http://www.phenoscanner.medschl.cam.ac.uk/) to find traits directly related to significant targets and delete related SNPs [30].

### 2.7 Colocalization analysis

We used the coloc R package for colocalization analysis [31], testing whether the significant targets and tinnitus had been determined to be associated with linkage disequilibrium. Bayesian methods evaluated five exclusive hypotheses: 1) not associated with either trait; 2) only associated with trait 1; 3) only associated with trait 2; 4) both traits are correlated, but have different causal variants; 5) both traits are correlated, and both traits have causal variants. The analysis provided posterior probabilities for each hypothesis test (H0, H1, H2, H3, and H4). Using the coloc.abf algorithm, evidence for colocalization was defined as a posterior probability of greater than 0.8 for shared causal variation (posterior probability of hypothesis PH4>0.8).

### 2.8 Molecular docking

Based on the above results, the top three associated active ingredients in “active ingredient-target” were selected as ligands, and the top 5 associated targets suggested by the MCC algorithm and significant in MR analysis were selected as receptor molecules for molecular docking. The target protein structure was searched from the Protein Data Bank (PDB) (https://www.rcsb.org/) [32]. The PDB is a public protein structure database that contains a large number of experimentally determined biological macromolecular structures. And the three-dimensional structure of active ingredients downloaded from PubChem CID (https://pubchem.ncbi.nlm.nih.gov/) and uniport databases was imported into AutoDockVina and PyMoL software [33] to find the best conformation. AutoDockVina molecular docking is an open source tool, widely used for predicting small molecule and protein patterns and affinity. PyMoL is a molecular visualization tool that allows us to perform detailed examination and analysis of molecular docking results. Visualization of docking results is essential for understanding the interaction between ligands and receptors. To this end, we further use the Universitat Hamburg provided ProteinsPlus PoseView tools [34] of the server. PoseView provides a user-friendly interface, used for analysis and present the results of molecular docking, including hydrogen bonds, hydrophobic interactions and other key role.

### 2.9 Molecular dynamics simulation

We refer to the method of Zhao et al. to perform Molecular Dynamics Simulations (MDs) [35]. We use the Generalized AMBER force field (GAFF) in the Antechamber of AMBER 18 to construct topology files for Quercetin, Kaempferol, and Wogonin. Subsequently, we establish the topology file for HIF1A using the Amber99sb-ildn in the GROMACS 19.5 software package (https://manual.gromacs.org/) and combine it with the ligand topology file to form the protein-ligand complex topology file. The Molecular Dynamics Simulations for the individual protein and the protein-ligand complex are carried out in the GROMACS 19.5 software package, using TIP3P as the solvent under periodic boundary conditions in a cubic box, and adding 68 Na+ and 59 Cl-as needed to neutralize the charge. The energy is optimized using the steepest descent method to ensure the energy is less than 1000.0 kJ/mol/nm. After energy optimization, 1 ns of constant temperature and volume (NVT) and 2 ns of constant temperature and pressure (NPT) are used to ensure that the system is maintained at a constant temperature (310.15 K) and pressure (0.1 MPa) for 100 ns MDs, which are accelerated using NVIDIA GeForce RTX 3080.

We extract the XTC file of the stable complex from 90 to 100 ns of the protein-ligand complex and calculate the binding free energy between the ligand and the receptor using the Molecular Mechanics Generalized Born Surface Area (MM/GBSA) method with gmx_MMPBSA [36]. This study also uses GMX commands to extract Root Mean Square Deviation (RMSD), Radius of Gyration (Rg), Solvent Accessible Surface Area (SASA), Root Mean Square Fluctuation (RMSF), and the number of hydrogen bonds between the ligand and the receptor for further analysis.

## 3 Results

### 3.1 Network analysis of xiaochaihutang prescription

**Fig. 2:**
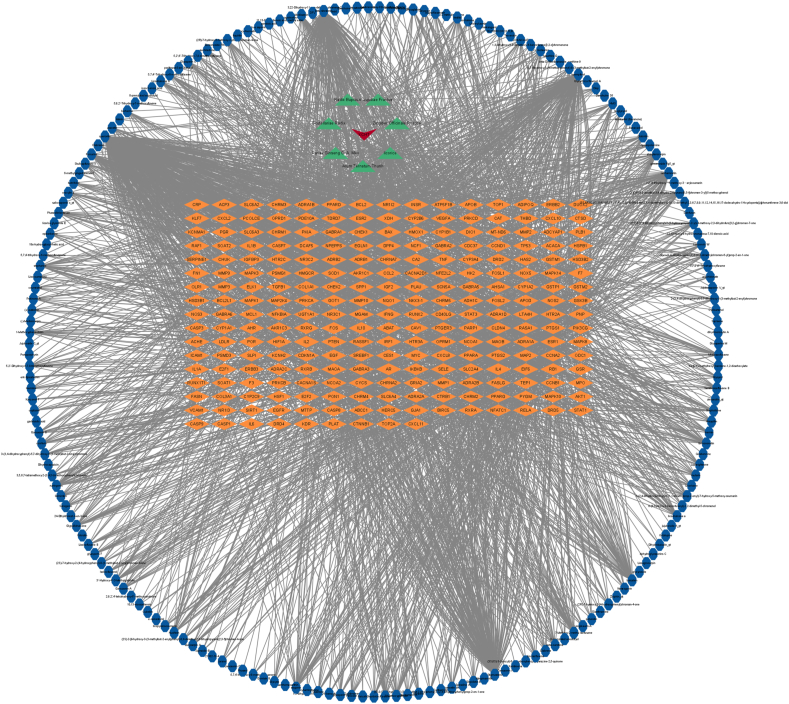
Prescription-Herbal-ingredient-target network diagram.V is Prescription, triangle is Herbal, octagon is ingredient,diamond is target. The formula-TCM composition-target network diagram is used to describe the relationship between TCM drug components and their targets. In the network diagram, Chinese medicine is usually represented as a node that represents the entity of the entire Chinese medicine. The drug ingredient is represented by another node, which represents the active ingredient in the traditional Chinese medicine. The action target is represented as the third node, which represents the action target of the drug ingredient in the biological body, such as receptors, enzymes, etc. The connecting lines between these nodes represent the interaction between the components of a traditional Chinese medicine and its target. By analyzing the network diagram, we can reveal the complex interaction between the components of traditional Chinese medicine and the target of action, and provide important reference for the research and development of traditional Chinese medicine.

The active ingredients of XCHT were identified from the TCMSP database, resulting in a total of 249 effective targets after removing duplicates(supplementary materials 1). Additionally, 391 potential targets related to tinnitus were collected from the GeneCards database(supplementary materials 2). After removing duplicates and selecting targets with a relevance score >1, a network analysis diagram of XCHT prescription, herbal ingredients, and their corresponding targets was constructed using Cytoscape software (Fig. 2).

### 3.2 Venn diagram analysis

**Fig. 3:**
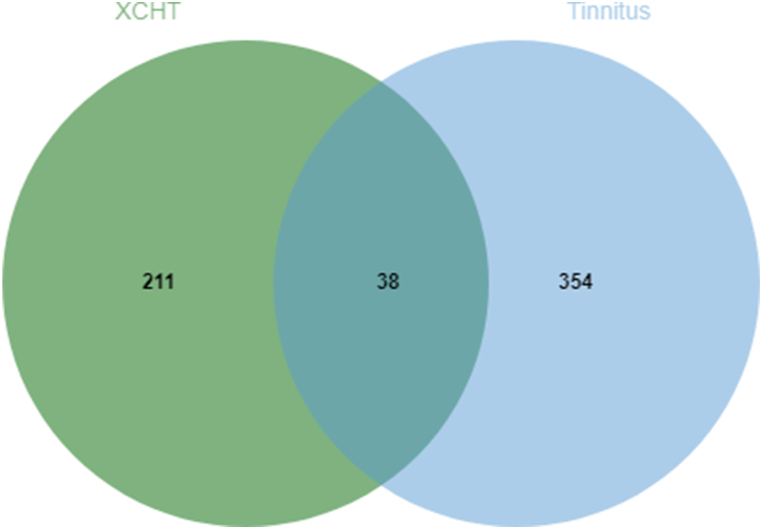
Venn diagram of Xiaochaihutang and Tinnitus.

By matching the effective ingredient targets of XCHT with potential targets of tinnitus, a total of 38 overlapping targets were obtained. A Venn diagram was generated to illustrate these overlapping targets, suggesting they may be crucial targets for XCHT in treating tinnitus (Fig. 3).

### 3.3 Protein-protein interaction network analysis

**Fig. 4:**
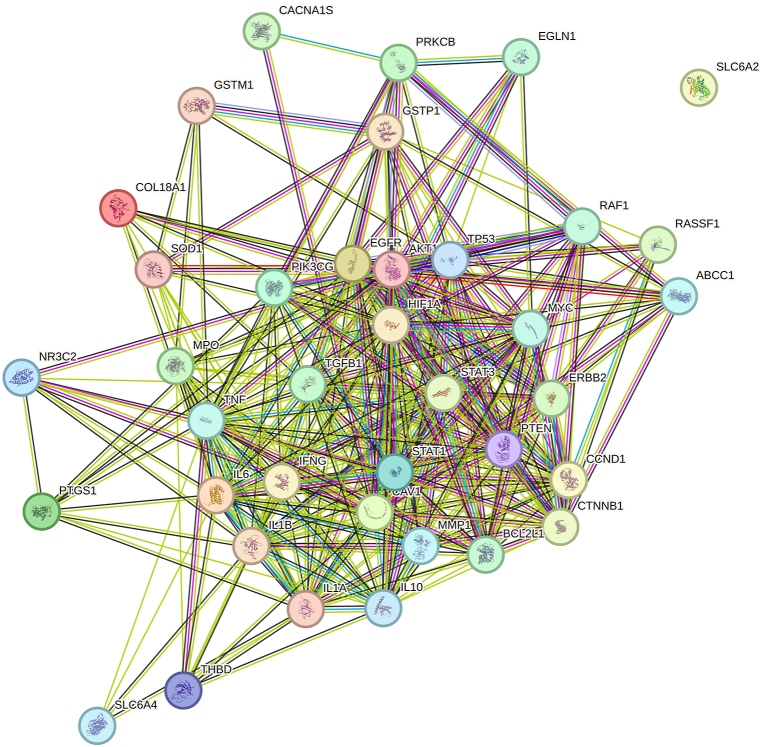
Xiaochaihutang-Tinnitus PPI network.

Using the String website, a PPI network diagram of the 38 overlapping targets was obtained (Fig. 4)(supplementary materials 3). Further analysis in Cytoscape software revealed the degree of correlation among the overlapping targets, identifying the top 5 core targets: AKT1, TP53, HIF1A, ERBB2, and STAT3.

### 3.4 Mendelian randomization analysis

**Fig. 5:**
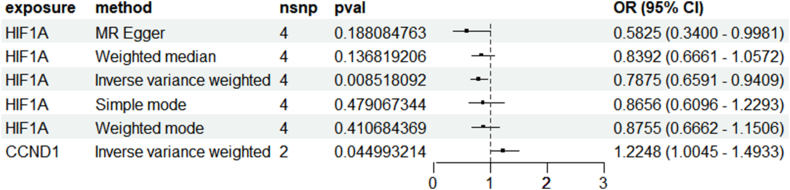
Forest plot of the relationship between significant genes and the risk of tinnitus onset by Mendelian randomization analysis.

**Fig. 6:**
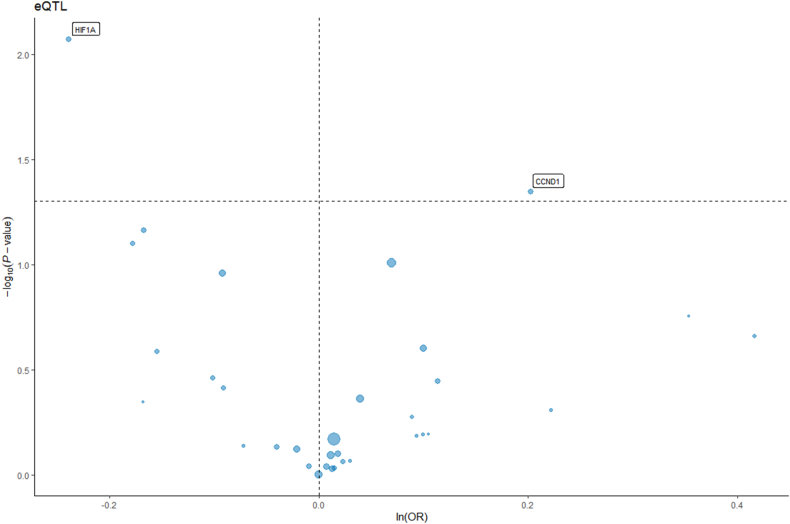
Volcano plot illustrating the association of significant genes with tinnitus risk.

After screening, a total of 128 SNPs associated with tinnitus were included in the study(supplementary materials 4). MR analysis indicated a significant association between HIF1A and CCND1 with tinnitus. HIF1A may act as a protective target for tinnitus, while CCND1 may promote its occurrence and development. The results showed significant associations of HIF1A (OR [95 %] = 0.78 [0.65, 0.94], p = 0.008) and CCND1 (OR [95 %] = 1.22 [1.00, 1.49], p = 0.04) with tinnitus in MR analysis IVW results (Fig. 5, Fig. 6).

### 3.5 Colocalization analysis

**Fig. 7:**
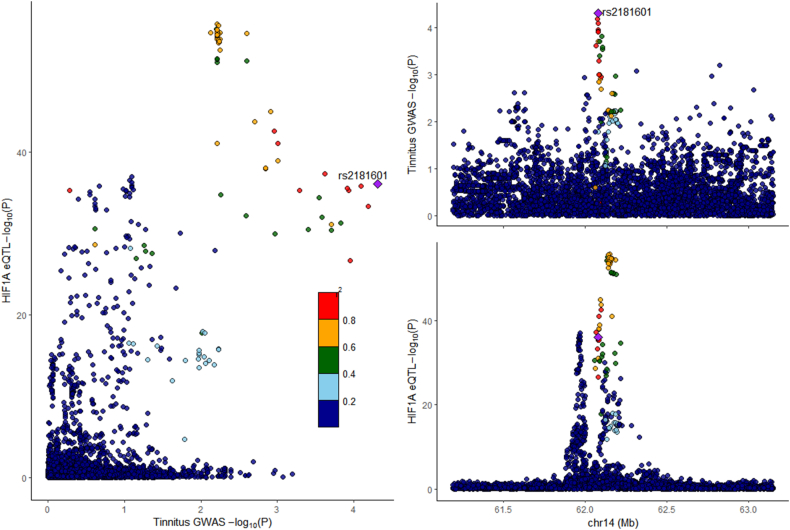
Regional association map: HIF1A and tinnitus.

**Fig. 8:**
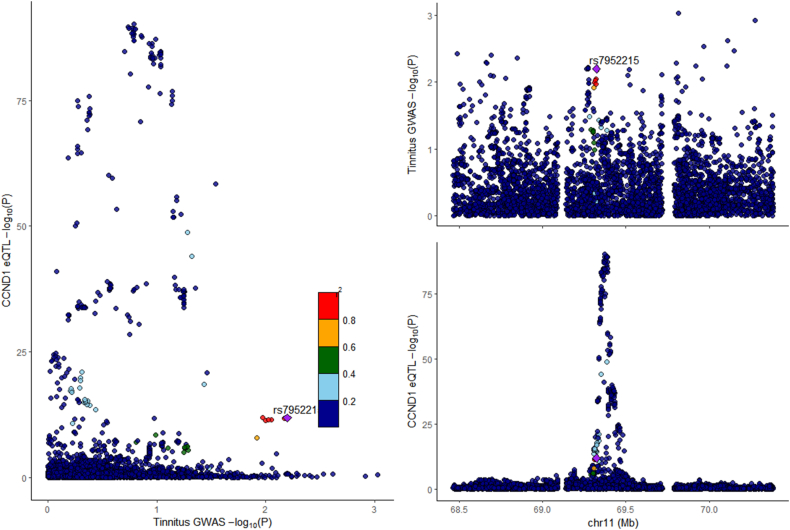
Regional association map: CCND1 and tinnitus.

Colocalization analysis of HIF1A and CCND1 with tinnitus indicated no shared colocalization for both HIF1A (PH4 = 0.34) and CCND1 (PH4 = 0.02) (Fig. 7, Fig. 8).

### 3.6 Molecular docking validation

**Fig. 9:**
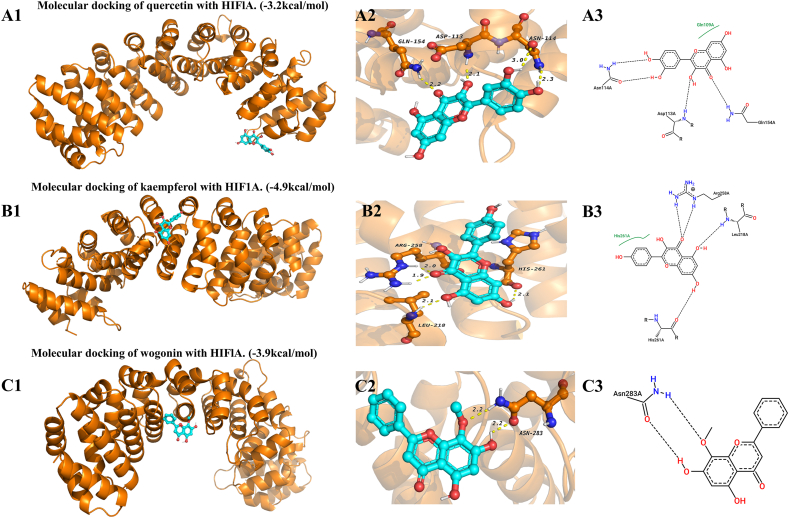
Molecular docking diagram of HIF1A with quercetin, kaempferol, and wogonin. Panel A illustrates HIF1A and quercetin with a binding energy of −3.2 kcal/mol, Panel B shows HIF1A and kaempferol with a binding energy of −4.9 kcal/mol, and Panel C depicts HIF1A and wogonin with a binding energy of −3.9 kcal/mol. Numbers 1 to 3 represent the binding site diagram between the ligand and HIF1A, the 3D hydrogen bond diagram, and the interaction force diagram, respectively. In number 3, the dashed lines represent hydrogen bonds between the ligand and key amino acid residues, and the green lines represent hydrophobic interaction forces. (For interpretation of the references to colour in this figure legend, the reader is referred to the Web version of this article.)

Using AutoDockVina1.1.2 and PyMoL software, molecular docking was performed between the top 3 active ingredients (quercetin, kaempferol, wogonin) and the top 5 targets (including HIF1A) suggested by the MCC algorithm and significant in MR analysis. The results indicated strong binding between these active ingredients and targets, with binding energies below −1.2 kcal/mol (Fig. 9).

### 3.7 Molecular dynamics simulation

**Fig. 10:**
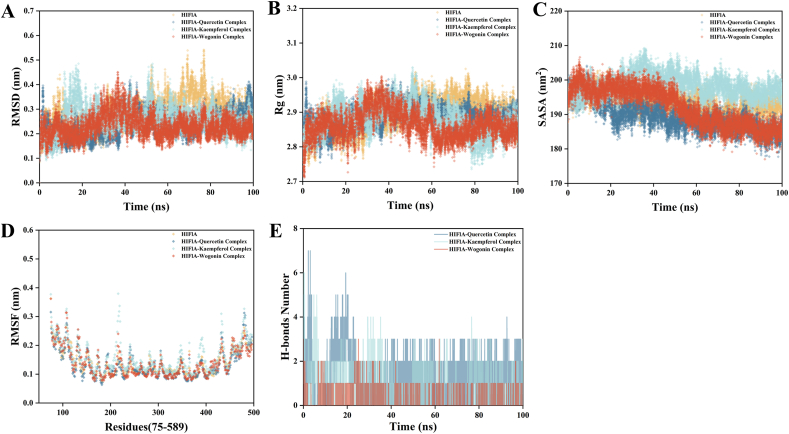
Molecular dynamics simulation results of small molecule-HIF1A complexes and individual HIF1A. In the figure, Panels A–D represent the Root Mean Square Deviation (RMSD), Radius of Gyration (Rg), Solvent Accessible Surface Area (SASA), and Root Mean Square Fluctuation (RMSF) of the small molecule-HIF1A complexes and individual HIF1A, respectively. Panel E represents the number of hydrogen bonds formed between Quercetin, Kaempferol, and Wogonin with HIF1A during the 100 ns Molecular dynamics simulation process.

The molecular dynamics simulation results (Fig. 10) indicate that the isolated HIF1A exhibits higher RMSD values during the simulation process. Upon binding with the ligand, the RMSD values of HIF1A decrease, suggesting that the ligand binding may enhance the stability of the protein. Additionally, the simulation results for isolated HIF1A suggest relatively larger Rg values, indicating that the protein is more loosely packed in the absence of the ligand, and higher SASA values, indicating that more surface areas are exposed to the solvent. After ligand binding, the RMSF values of the regions directly interacting with the ligand decrease, indicating a reduction in the dynamics of these regions and a more stable local structure of the protein. The number of hydrogen bonds formed between Quercetin and HIF1A remains relatively stable throughout the simulation process, showing a strong interaction. The number of hydrogen bonds formed between Kaempferol and HIF1A is lower, indicating a weaker interaction. Wogonin forms the highest number of hydrogen bonds with HIF1A, showing the strongest interaction. This suggests that the binding of the ligand significantly affects the stability and dynamics of HIF1A, and the interaction between the ligand and HIF1A enhances the protein's stability and limits its dynamics.(supplementary materials 5)

The MM-PBSA results (Table 1) show that the binding free energies of Quercetin, Kaempferol, and Wogonin with HIF1A are −37.06, −19.23, and −83.36 kJ/mol, respectively, indicating that Wogonin binds most stably with HIF1A. The polar solvation free energy value of the Wogonin-HIF1A complex is the highest, suggesting that it has the highest stability in aqueous solution. The interaction value between the Wogonin-HIF1A enzyme-substrate complex and the solvent is the lowest, indicating the least interaction with the solvent. The Wogonin-HIF1A complex shows strong interactions in both Coulombic energy and van der Waals interaction energy. These results suggest that the binding of the ligand significantly alters the energy parameters of HIF1A, particularly the binding free energy and solvation free energy, which allows us to better understand the thermodynamic properties of the interaction between the ligand and HIF1A.(supplementary materials 6)

**Table 1:** Energy Decomposition Results (KJ/mol) Calculated by MM-PBSA Method between Quercetin, Kaempferol and Wogonin and HIF1A respectively.

Complex	MM-PBSA (KJ/mol)				
	E_Binding_	E_PB_	E_SASA_	E_Coul_	E_Vdw_
Quercetin-HIF1A	−37.06 ± 6.66	75.48 ± 7.10	−12.87 ± 0.24	−7.81 ± 5.23	−91.86 ± 4.95
Kaempferol-HIF1A	−19.23 ± 3.67	41.00 ± 2.22	−4.39 ± 0.49	−2.86 ± 1.01	−78.39 ± 3.94
Wogonin-HIF1A	−83.36 ± 8.49	74.67 ± 13.38	−16.61 ± 0.37	−27.83 ± 4.33	−113.59 ± 6.67

Table notes: E_Binding_: binding free energy between small molecule and protein, E_PB_: polar solvation free energy, E_SASA_: interaction of enzyme substrate complex with solvent, E_Coul_: Coulomb energy, E_Vdw_: van der Waals interaction energy.

## 4 Discussion

In this study, through network pharmacology analysis, we identified 249 effective component targets of XCHT and 391 potential targets for tinnitus, with 38 overlapping targets between the two. Subsequently, through Mendelian randomization analysis, we found that HIF1A and CCND1 are significantly associated with tinnitus disease. The results suggest that HIF1A has a potential protective role in the development of tinnitus, while CCND1 has a promoting effect. Among them, HIF1A is one of the significantly related targets to tinnitus and is also among the top five key nodes in terms of association degree. In molecular docking and molecular dynamics simulations, we found that HIF1A has a good binding effect with the active components of XCHT, such as quercetin, kaempferol, and wogonin. Therefore, we believe that HIF1A may play a key role in the treatment of tinnitus by XCHT.

The mechanism of XCHT in treating tinnitus involves several aspects. Studies have shown that the herbal compound XCHT, which includes the herb Radix Bupleuri, may help restore the body's state balance and alleviate symptoms associated with this condition. This effect is thought to be mediated through its ability to modulate the body's stress response and promote overall well-being [37]. Scutellaria baicalensis and Ginseng are recognized for their anti-inflammatory and detoxifying effects. These properties may help to reduce the body's inflammatory response, which is often associated with the perception of tinnitus. By addressing underlying inflammation, these herbs may contribute to the mitigation of tinnitus symptoms [38]. Pinellia, a component of XCHT, has been traditionally used to alleviate symptoms such as phlegm production and nausea. Its calming effects on the nervous system may contribute to the relief of tinnitus symptoms by reducing stress and promoting relaxation [39]. Additionally, Ginseng and Jujube, key ingredients in XCHT, are believed to enhance vitality and support the circulatory system. Modern interpretations suggest that these herbs may help to improve overall energy levels and support healthy blood flow, which could potentially alleviate symptoms of tinnitus by promoting better nutrient delivery and reducing stress on the auditory system [40]. Licorice, another ingredient in XCHT, is recognized for its potential to support the body's energy levels, enhance circulation, and support digestive health. These effects may be beneficial in managing the symptoms of tinnitus by improving overall systemic health and reducing stress-related symptoms [41]. The therapeutic effects of XCHT in managing tinnitus are believed to operate through a multifaceted approach that addresses various physiological imbalances. This includes modulating the body's stress response. Additionally, the formula is thought to reduce inflammation and support detoxification processes. Furthermore, it is suggested that XCHT may enhance overall vitality and support the circulatory system. These combined actions are intended to alleviate the symptoms of tinnitus and contribute to the restoration of auditory comfort.

Currently, there is no direct evidence indicating a direct association between XCHT and HIF1A. However, the main constituents of XCHT are believed to be associated with HIF1A. For example, studies have shown that Radix Bupleuri extract inhibits the expression of Bax, Caspase-3, and Caspase-9 through the IL-6/HIF1A signaling pathway, and regulates malondialdehyde, superoxide dismutase, and glutathione peroxidase [42]. Additionally, studies have demonstrated that baicalein, a natural flavonoid compound found in Scutellaria baicalensis, can reduce the expression levels of HIF1A and VEGF to exert antioxidant effects and alleviate PC12 cell hypoxia-induced injury. Furthermore, experiments conducted by Li have shown that active ingredients in Ginseng, such as ginsenoside Rg3, reduce the expression of EGF, EGFR, phosphorylated ERK1/2, and HIF1A. Moreover, some studies have suggested that XCHT can affect cell metabolism and survival through pathways such as immune regulation, anti-inflammatory, and antioxidant mechanisms [[43], [44], [45]], while the HIF1A protein also plays a role in regulating cell metabolism and survival [46,47]. Therefore, XCHT may exert its pharmacological effects by influencing the expression or activity of the HIF1A protein, but the specific molecular mechanisms require further elucidation.

Currently, there is also no direct evidence indicating a direct association between HIF1A and tinnitus. However, some studies have suggested that hypoxia may be associated with tinnitus [48], and HIF1A, as a protein that regulates cellular responses to hypoxia [49], may influence the occurrence of tinnitus to some extent. HIF1A may significantly inhibit the occurrence and development of tinnitus. HIF1A, an important transcriptional regulator of cell survival under hypoxic conditions, plays a crucial role in angiogenesis [50,51]. Oxygen is crucial for tinnitus, and although the efficacy of hyperbaric oxygen therapy is currently unclear [52], some studies suggest that hyperbaric oxygen therapy can improve tinnitus symptoms by improving cochlear blood supply and metabolism in a high-pressure oxygen environment [53,54]. The action of HIF1A enables patients to protect auditory cells as much as possible under low oxygen conditions, thereby reducing tinnitus. However, further research is needed to determine the exact relationship between HIF1A and tinnitus, as well as the potential role of HIF1A in tinnitus treatment.

This study is the first to use a combination of network pharmacology, MR, and molecular docking methods, and molecular dynamics simulation to explore the potential targets of TCM for disease action. Through network pharmacology and molecular docking, we explored the synergistic effects of TCM multi-components, multi-channels, and multi-targets. Through MR, we clarified the significant association between potential targets and diseases and excluded the interference of confounding factors on the results. This is more conducive to our subsequent exploration of the mechanism of action and potential targets of XCHT in the treatment of tinnitus. However, this study still has certain limitations as all our research data come from databases, and therefore, the reliability and accuracy of the predictions depend on the quality of the data. In the future, we need to further utilize clinical trials and animal experiments to validate our results.

## 5 Conclusion

This study explores the mechanism of XCHT in treating tinnitus using network pharmacology, MR analysis, molecular docking, and molecular dynamics simulations. Our results indicate that quercetin, kaempferol, and wogonin are key bioactive compounds in XCHT for treating tinnitus. Furthermore, through MR analysis, HIF1A was identified as a potential protective factor, significantly negatively correlated with the risk of tinnitus (OR = 0.78, p = 0.008), while CCND1 showed a positive correlation (OR = 1.22, p = 0.04). Molecular docking studies suggest that quercetin, kaempferol, and wogonin have strong binding affinity with HIF1A, with binding energies of −3.2, −4.9, and −3.9 kcal/mol, respectively, indicating strong interactions with the HIF1A target. Molecular dynamics simulations further confirmed this interaction, showing a decrease in RMSD after HIF1A binding with ligands, indicating enhanced protein stability. Among them, wogonin showed the most significant stabilizing effect. Through MM-PBSA calculations, the binding free energy of wogonin was −83.36 kJ/mol, indicating that wogonin has good stability and strong intermolecular interactions. These results suggest the potential of XCHT in the treatment of tinnitus and provide a basis for further exploration of its molecular targets.

These findings provide new insights into the therapeutic mechanisms of the TCM formula XCHT for tinnitus and offer important references for further research and clinical applications. However, despite the enlightening and guiding significance of the results, there are limitations to this study that require further experimental validation and clinical research for confirmation. With continued research into XCHT, it is believed that better utilization of TCM for treating tinnitus and other related conditions can be achieved, benefiting more patients in the process. However, the specific mechanism and effect need to be further studied and verified.

## Funding statement

Supported by the Medical Science and Technology Research Program of Chongqing Banan Science and Technology Bureau and Chongqing Banan Health Commission, Grant/Award Number: BNWJ202300135.

## Ethics approval and consent to participate

Not applicable.

## Consent for publication

Not Applicable.

## Data availability

All the data can be obtained from the open source platform provided in the article. All data were obtained from public databases.

## CRediT authorship contribution statement

**Shihan Liu:** Writing – original draft, Validation, Investigation, Formal analysis, Conceptualization. **Xintong Zhou:** Writing – review & editing, Validation, Methodology, Formal analysis. **Lingli Zhang:** Writing – review & editing, Supervision, Project administration, Conceptualization. **Wenlong Luo:** Writing – review & editing, Methodology.

## Declaration of competing interest

The authors declare that they have no known competing financial interests or personal relationships that could have appeared to influence the work reported in this paper.
